# Estimating the differences in Caesarean section (C-section) rates between public and privately insured mothers in Florida: A decomposition approach

**DOI:** 10.1371/journal.pone.0266666

**Published:** 2022-04-07

**Authors:** Neeraj Puro, Reena J. Kelly, Mandar Bodas, Scott Feyereisen

**Affiliations:** 1 Department of Management Programs, College of Business, Florida Atlantic University, Boca Raton, Florida, United States of America; 2 Department of Health Administration and Policy, School of Health Sciences, University of New Haven, West Haven, CT, United States of America; 3 Fitzhugh Mullan Institute for Health Workforce Equity, The George Washington University Milken Institute School of Public Health, Washington, DC, United States of America; University of Georgia, UNITED STATES

## Abstract

**Background:**

Caesarean section (C-sections) is a medically critical and often life-saving procedure for prevention of childbirth complications. However, there are reports of its overuse, especially in women covered by private insurance as compared to public insurance. This study evaluates the difference in C-Section rates among nulliparous women in Florida hospitals across insurance groups and quantifies the contribution of maternal and hospital factors in explaining the difference in rates.

**Methods:**

We used Florida’s inpatient data provided by the Florida Agency for HealthCare Administration (FLAHCA) and focused on low-risk births that occurred between January 1, 2010, and September 30, 2015. A Fairlie decomposition method was performed on cross-sectional data to decompose the difference in C-Section rates between insurance groups into the proportion explained versus unexplained by the differences in observable maternal and hospital factors.

**Results:**

Of the 386,612 NTSV low-risk births, 72,984 were delivered via C-Section (18.87%). Higher prevalence of C-section at maternal level was associated with diabetes, hypertension, and the expectant mother being over 35 years old. Higher prevalence of C-section at the hospital level was associated with lower occupancy rate, presence of neonatal ICU (NICU) unit and higher obstetrics care level in the hospital. Private insurance coverage in expectant mothers is associated with C-section rates that were 4.4 percentage points higher as compared to that of public insurance. Just over 33.7% of the 4.4 percentage point difference in C-section rates between the two insurance groups can be accounted for by maternal and hospital factors.

**Conclusions:**

The study identifies that the prevalence of C-sections in expectant mothers covered by private insurance is higher compared to mothers covered by public insurance. Although, majority of the difference in C-Section rates across insurance groups remains unexplained (around 66.3%), the main contributor that explains the other 33.7% is advancing maternal age and socioeconomic status of the expectant mother. Further investigation to explore additional factors that explain the difference needs to be done if United States wants to target specific policies to lower overall C-Section rate.

## Introduction

Caesarean section (C-section) birth rates across the globe have doubled over the past two decades, eliciting concerns regarding C-section overuse [1]. According to the U.S. Centers for Disease Control and Prevention (CDC), in 2017, the C-section rate across the country was 32%, well in excess of the recommended rate of 10–15% [2]. Unnecessary C-sections not only put expectant and new mothers at risk of developing surgical complications and future reproductive challenges but have also been linked to autism spectrum and hyperactivity disorders in the offspring [3–5]. In addition to increased risks to maternal and infant health, C-sections also contribute to increased healthcare costs, with C-section births costing nearly twice as much as vaginal births [6].

Several factors have been linked to high C-section rates, including external factors such as the county where the expectant mother resides and delivers, the hospital in which the newborn is delivered, maternal insurance status, provider preferences, and frequency of malpractice suits [6–9]. While medical necessity questions still remain, in most cases, maternal age, clinical conditions such as diabetes, hypertension, hemorrhage, fetal complications, and psychosocial conditions, including depression and/or anxiety, are more likely to necessitate C-sections in expectant mothers [10, 11]. One important, yet relatively under researched factor in this context, is the impact of expectant mothers’ insurance status on C-section rates. Insurance status, and particularly the type of insurance coverage an expectant mother has, can be an important factor influencing the use of C-sections [12]. In general, there exist two main types of insurance coverage for expectant mothers: publicly funded programs such as Medicaid and CHIP, and private health insurance (employer-sponsored or marketplace plans under the ACA) [13].

Although plans are similar in the type of prenatal and obstetric care covered, reimbursement rates offered by insurers for C-sections tend to be higher than those for vaginal delivery [14]. Researchers have argued that financial incentives by payers influence supplier behavior (either hospitals or individual physicians), resulting in variations in C-section rates. Gruber et al. (1999) found that higher fee differentials between C-sections and vaginal deliveries resulted in higher C-section use for privately insured patients [15]. More recently, the Hoxha et al. (2017) review examined financial incentives associated with private insurance that may encourage healthcare providers to perform more C-sections [12]. Their study estimated that the overall odds of receiving a C-section were higher for privately insured women when compared to women covered by public insurance. Although these studies focused on the differences in C-section rates across insurance types, they did not necessarily control for hospital-level characteristics that might contribute to such differences. Kozhimannil et al. (2014) extended this research to include different individual- and hospital-level factors associated with variation in C-section rates across insurance coverage, but only used a limited number of hospital-level characteristics [16]. Despite the robust literature establishing that C-sections tend to be more common among privately insured women, to our knowledge, no study has quantified the differences in rates between the two insurance groups based on relevant individual and external characteristics, especially hospital-level factors, that might affect C-section rates. Given the central role of hospitals in influencing these outcomes, this is an important oversight. Our data and analytical strategy allow us to address this gap.

Our analysis is guided by two research questions: 1) Does the likelihood of receiving a C-section vary based on the mother’s insurance coverage? 2) If yes, how much of these differences are explained by individual (sociodemographic and clinical) and external factors (hospital-level)? This research is timely and critical for US policy development. For example, one of the three specific targets of the US Department of Health and Human Services’ (HHS) Action Plan is to improve the nation’s maternal health outcomes by 2025, including a 25% reduction in low- risk C-section deliveries [17]. A key step in achieving this goal is identifying the differences in C-section rates between Medicaid and private insurance recipients, and quantifying the differences attributable to important individual and hospital-level factors. Depending on the nature of the variation and the factors contributing most to it, there may be an opportunity for developing focused public policy aimed at minimizing unnecessary C-sections, thereby mitigating some of the associated negative consequences.

## Methods

### Data

We used Florida’s inpatient data provided by the Florida Agency for HealthCare Administration (FLAHCA), and focused on births that occurred between January 1, 2010, and September 30, 2015. The FLAHCA inpatient data includes all patients admitted to hospitals in Florida, but not patients admitted to non-ambulatory birth centers. Each patient level observation from FLAHCA includes identifiers for a hospital, as well as the patient’s demographic and health characteristics, such as insurance status, age, race, and health status, with up to 30 clinical diagnoses. We limited our sample to this time period since ICD version of the International Statistical Classification of Diseases and Related Health Problems (ICD) was changed from ICD-9 to ICD-10 after September 2015. Over the 6-year period (5 years and 9 months), there were 1,191,583 live births to Florida residents who delivered in Florida’s nonmilitary hospitals with maternity service. To reduce heterogeneity of the sample and to account for the confounding relationship between history of clinically complex pregnancies and C-sections, we focus solely on Nulliparous, Term, Singleton, Vertex (NTSV) low risk births with no history of C-section. We defined low risk vs high risk for C-section based on a manual published by Society for Maternal-Fetal Medicine (SMFM) in 2015. The SMFM definition is based solely on diagnoses identifiable in administrative claims-based data described by the International Classification of Diseases, Ninth Revision (ICD-9), Clinical Modification (ICD-9-CM) [18, 19]. To identify patients who had a C-section birth, we used the assigned diagnosis-related group (DRG) codes: DRG-765 and DRG-766.

**Fig 1:**
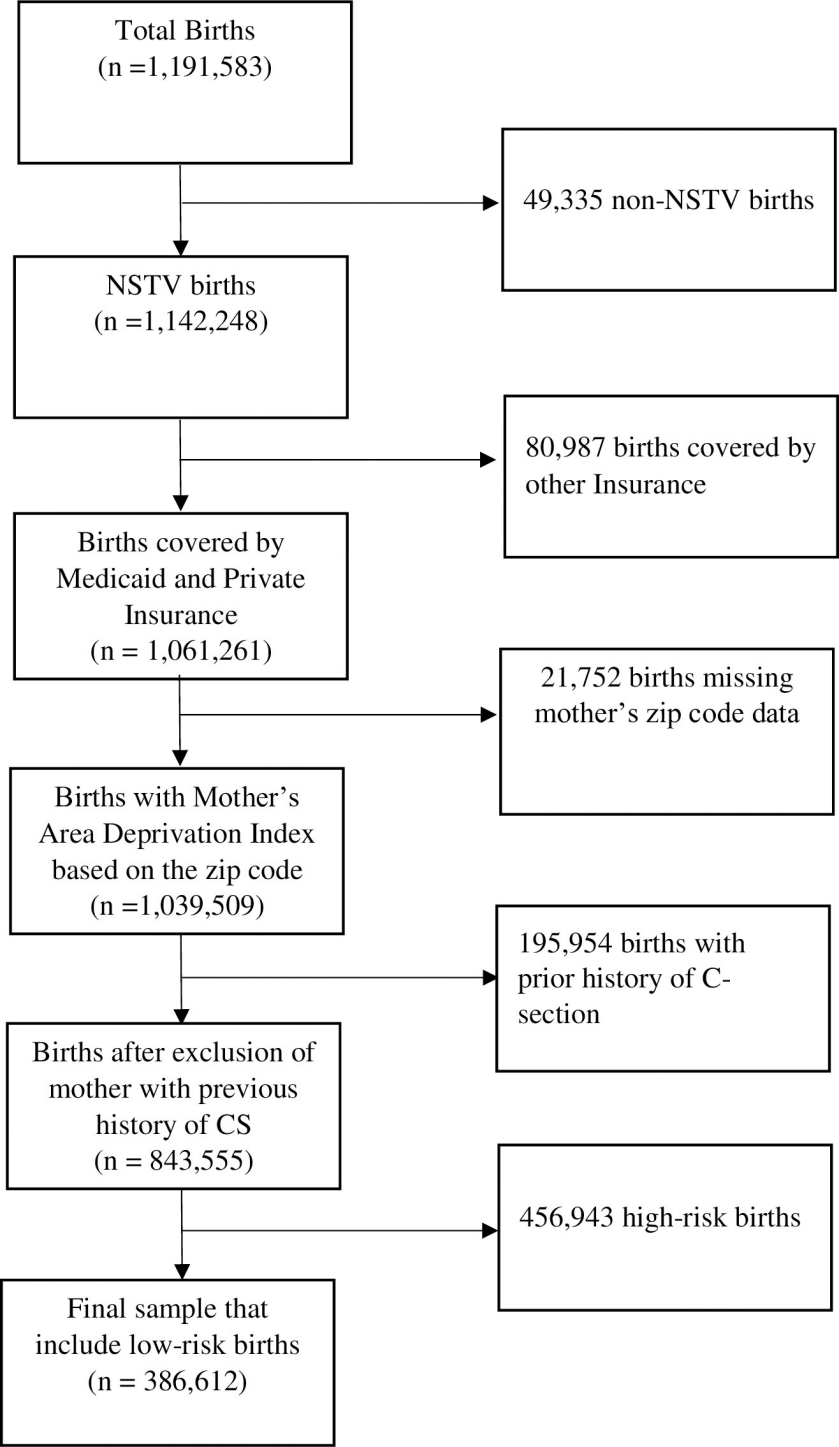
Flow chart for selecting the analytical sample (N = 386,612).

The flow chart for selecting the analytical sample is presented in, “Fig 1”. Of the total 1,191,583 births, we excluded 130,322 births (49,335 non-NSTV births and another 80,987 births covered by other Insurance) restricting sample to only NSTV births covered by either Medicaid or private insurance leading to a sample size of 1,061,261 births. We then used the zip code of maternal residence to merge the inpatient data with Area Deprivation Index data to obtain sociodemographic characteristics of maternal area of residence. The linkage led to exclusion of an additional 21,752 births that had missing zip codes. Finally, we excluded 195,954 mothers with previous history of C-sections resulting in a sample of 843,555 births. Along with sociodemographic and clinical characteristics, we also added hospital characteristics to create the complete dataset. Using hospital identifier of FLAHCA, we merged the inpatient data with American Hospital Association (AHA) data to obtain hospital characteristics. From the final number of 843,555 births (high risk and low risk), we excluded high risk births and those observations with missing hospital variables leading to a final sample size of 386,612 births.

### Measurement

#### Dependent variables

The dependent variable is a binary indicator of whether a NTSV mother had a C-section delivery or not.

#### Independent variables

The main independent variable of interest was type of maternal insurance, specifically whether the mother was covered by a private insurance or Medicaid. We included sociodemographic factors such as gestational age, race, and socioeconomic status (SES). SES was measured by level of Area Deprivation Index (ADI), divided into quartiles, obtained based on the zip code of the mother’s residence [20]. Area Deprivation Index (ADI) is composed of socioeconomic variables including median family income, family poverty rate, income disparity, occupational composition, percentage of population below 150% of poverty rate, single parent household rate, home ownership rate, median home value, median gross rent, household crowding, median monthly mortgage, and educational distribution. Other variables that are also included are percentage of households without access to telephones, plumbing, or motor vehicles; percentage of urban population; percentage of immigrant population; English language proficiency; and divorce rates.

We controlled for individual clinical factors such as hypertension, Type II diabetes, asthma, substance use disorders, and infections. In addition to the individual-level factors, we also included various hospital-level characteristics that could influence probability of a mother getting a C-section, including hospital occupancy rate, teaching status, system membership, ownership, presence of neonatal intensive care unit (NICU), and the level of obstetric care offered. Occupancy rate was measured as a ratio of total inpatient days to the total number of beds in a calendar year. We included a categorical variable for hospital ownership such that ownership was “0” if it had not-for-profit status, “1” if it had nonfederal government status, and “2” if it was a for-profit hospital. System membership was included as a binary indicator of whether the hospital was part of a larger health system or not. A hospital was considered a teaching hospital if it was a member of the Council of Teaching Hospitals and Health Systems. To capture the presence of a NICU in the hospital, we included a dichotomous indicator where the variable was “0” if the hospital did not have a NICU, and “1” if it had NICU. The level of obstetrics care was divided into 3 categories as measure by American Hospital Association (AHA) Annual Survey of Hospitals.

### Analytic strategy

We first analyze determinants of C-section using variables chosen for the multivariate logistic regression analysis based on extant literature [16, 21] (Table 2). We tested for multicollinearity, outliers, and linearity to ensure model fit for the following logistic regression model.

$$Y_{i}=F(X_{i}\beta )$$

where Y_i_ is the dichotomous variable which indicates whether the woman had a C-Section and X_i_ is a vector of individual- and hospital-level factors.

Next, the dependent variable being binary, we used a non-linear approximation of the Blinder-Oaxaca decomposition technique to examine sociodemographic, clinical and hospital-level factors contributing to C-sections (Table 3) [22]. We analyzed the same variables in the logistic regression except for insurance, which became the basis for comparison. The Blinder-Oaxaca decomposition analysis has been used to ascertain the contributions of underlying factors in racial/ethnic differences in the outcome variables that include low birth weight, child mortality, breastfeeding and C-section [23–26]. In our study, we apply this technique to decompose the mean differential of C-section relative to vaginal births between public (Medicaid) and private insurance. The decomposition explains the proportion of sociodemographic, clinical and hospital-level differences in C-sections observed in the model (Table 3). The average difference in the C-section rates between publicly insured and privately insured mothers’ women may be expressed as:

Y¯pub−Y¯pri=[∑i=1NpubF(Xipubβ^pub)Npub−∑i=1NpriF(Xipriβ^pub)Npri]+[∑i=1NpriF(Xipriβ^pub)Npri−∑i=1Npri(2)F(Xipriβ^pri)Npri]

In the above equation, ${\bar{Y}}^{J}$ is the average probability of C-section for group J (J = pub,pri) $X_{i}^{J}$ is the vector of individual and hospital variables of observation *i* in group *J*, β^J is the vector of coefficient estimates and *N* ^*J*^ is the number of observations in group *J*. In this case, group *pub* is the sample of mothers covered by public insurance, group *pri* is the sample of mothers covered by private insurance, and the reference is group *pub*. Following the study by Brick et al (2016) [26], we also do the decomposition using the estimated coefficients of the pooled coefficients as the reference.

In this application, the first term on the right-hand side of [2] measures the amount of the gap in the C-Section rate that is due to differences in the characteristics of the two groups (public and private insurance in this case). The second term captures the degree to which mothers covered by public and privately insured mothers with similar observable characteristics have different C-Section rates. This may be interpreted as reflecting, varying hospital obstetric practice, or other omitted variables. We use the ‘Fairlie’ decomposition command in STATA 17 with randomly ordered variables and 1,000 replications [26].

### Ethical consideration

This is a retrospective study of medical records. The privacy, anonymity, and confidentiality of information were maintained in the process of data transfer from Agency for Healthcare Administration (AHCA) of Florida to the primary author. All medical record information were de-identified using unique alphanumeric codes generated by AHCA. Given the de-identified nature of the data, this study used secondary data for analysis and did not require IRB from the primary author’s institution.

## Results

Table 1 presents the descriptive statistics of the sample births (N = 386,612) included in this analysis. Overall, a greater proportion of privately insured mothers had a C-section compared to those covered by Medicaid (21.45% vs. 17.05%). Overall, 12.38% of the women had age 35 and above. The sample population consisted largely of the women with white as a race (65.17%). The C-section rate among Asian mothers covered by Medicaid was 14.78% compared to 21% covered by Private Insurance. The average occupancy rate of the hospitals in the sample was 66.11%. The population seems to be equally distributed in terms of socioeconomic status as measured by Area Deprivation Index. Approximately, 10% of the sample had hypertension as a clinical factor. Most of the hospitals in the sample had a not-for-profit status (65%).

**Table 1:** Descriptive statistics.

	Total (%)	Medicaid Coverage		Private Insurance Coverage (n = 160,237)	
		(n = 226,375)			
		Vaginal Births	Cesarean Births	Vaginal Births	Cesarean Births
N	386,612	187,777 (82.94%)	38,598 (17.05%)	125,851 (78.55%)	34,386 (21.45%)
***Sociodemographic factors***					
*Maternal age*					
<19 years	21,317 (5.51%)	15,294 (83.22%)	3,084 (16.78%)	2,416	523
				(82.20%)	(17.80%)
19–24 years	120,231 (31.10%)	78,516 (83.07%)	16,006 (16.93%)	20,457 (79.57%)	5,282 (20.43%)
25–29 years	88,347 (22.85%)	43,280 (84.45%)	7,967 (15.55%)	29,551 (79.65%)	7,549 (20.35%)
30–34 years	108,844 (28.15%)	36,634 (83.10%)	7,449 (16.90%)	51,548 (79.6%)	13,213 (20.4%)
>35 years	47,873 (12.38%)	14,053	4,092 (22.55%)	21,879 (73.6%)	7,849
		(77.45%)			(26.4%)
*Race*					
White	251,936 (65.17%)	108.088 (82.69%)	22,631 (17.31%)	95,708 (78.96%)	25,509 (21.04%)
Black	86,980 (22.5%)	54,718 (82.43%)	11,666 (17.57%)	15,695 (76.20%)	4,901
					(23.8%)
Asians	7,108	2,156 (85.22%)	374	3,605 (78.75%)	973
	(1.84%)		(14.78%)		(21.25%)
Native Americans/ Alaskan Natives	1,111	540	102	369	100
	(0.29%)	(84.11%)	(15.89%)	(78.68%)	(21.32%)
Others	39,477 (10.21%)	22,275 (85.34%)	3,825 (14.66%)	10,474 (78.3%)	2,903
					(21.7%)
Are Deprivation Index					
Area Deprivation index–Q_1_	92,524 (23.93%)	31,328 (80.28%)	7,696 (19.72%)	41,717 (77.98%)	11,783 (22.02%)
Area Deprivation index–Q_2_	100,013 (25.87%)	47,644 (82.58%)	10,051 (17.42%)	33,081 (78.17%)	9,237 (21.83%)
Area Deprivation index–Q_3_	93,691 (24.23%)	49,646 (83.73%)	9,645 (16.27%)	27,210 (79.10%)	7,190
					(20.9%)
Area Deprivation index–Q_4_	100.384 (25.97%)	59,159 (84.07%)	11,206 (15.93%)	23,843 (79.43%)	6,176 (20.57%)
***Clinical factors***					
Hypertension	38,759 (10.03%)	15,069 (8.02%)	6,910 (17.90%)	10,711 (8.51%)	6,069 (17.65%)
Diabetes	23,291 (6.02%)	9,455	3,771	6,732	3,333
		(5.04%)	(9.77%)	(5.35%)	(9.69%)
Asthma	12,355	6,701 (3.57%)	1,532 (3.97%)	3,077 (2.44%)	1,045 (3.04%)
	(3.2%)				
Substance use disorders	1,412	1,079 (0.57%)	199	105	29
	(0.37%)		(0.52%)	(0.08%)	(0.08%)
Infections	3,246	1,202 (0.64%)	632	865	547
	(0.84%)		(1.64%)	(0.69%)	(1.59%)
***Hospital factors***					
Occupancy Rate	66.11% (9.62%)	66.46% (9.5%)	65.99% (9.97%)	65.88% (9.64%)	65.19% (9.69%)
NICU Status	286,518 (74.11%)	131,128 (69.83%)	28,725 (74.42%)	98,537 (78.3%)	28,128 (81.8%)
Obstetrics care–Level 1	95,140 (24.61%)	50,797 (27.05%)	9,449 (24.28%)	28,246 (22.44%)	6,648 (19.33%)
Obstetrics care–Level 2	135,135 (34.95%)	57,247 (30.49%)	12,326 (31.93%)	51,620 (41.02%)	13,942 (40.55%)
Obstetrics care–Level 3	156,337 (40.44%)	79,733 (42.46%)	16,823 (43.59%)	45,985 (36.54%)	13,796 (40.12%)
System membership	260,956 (67.5%)	125,076 (66.61%)	27,027 (70.02%)	84,577 (67.2%)	24,276 (70.6%)
Teaching hospital	58,484 (15.39%)	35,789 (19.06%)	7,216	12,618 (10.03%)	3,861 (11.23%)
			(18.7%)		
Not-for profit hospitals	251,445 (65.04%)	112,575 (59.95%)	22,701 (58.81%)	90,879 (72.21%)	25,290 (73.55%)
For-profit hospitals	62,360 (15.13%)	34,111 (18.17%)	6,830	17,323 (13.76%)	4,096 (11.91%)
			(17.7%)		
Non-federal Government hospitals	72,807 (18.83%)	41,091 (21.88%)	9,067 (23.49%)	17,649 (14.02%)	5,000
					(14.54%)

### Determinants of C-section

The results of our logistic regression are presented in Table 2. We provide odds ratios and the corresponding 95% confidence intervals for the analysis establishing the association between respondent characteristics and odds of getting a C-section. Privately insured mothers had 1.23 higher odds of getting a C-section compared to those on Medicaid (p<0.01). The odds of getting a C-section were significantly higher for women older than 35 years compared to women aged 19–24 years. The odds of getting a C-section decreased with the decrease in socioeconomic status of the mother. In addition, the odds of black women getting a C-section were significantly higher compared to white women (OR = 1.03, p<0.01). The associations between the mother’s sociodemographic characteristics and the likelihood of getting a C-section were consistent with previous studies [16, 27]. Type II diabetes and hypertension were amongst the strongest individual-level clinical predictors of the likelihood of getting a C-section.

**Table 2:** Determinants of NTSV low-risk C-sections at hospital discharge, 2010–2015 (n = 386,612).

	Determinants of C-section		
	Estimate	95% CI	Sig
***Sociodemographic factors***			
Medicaid coverage	Referent	Referent	Referent
Private insurance coverage	1.24	(1.21,1.26)	***
Maternal age			
<19 years	0.97	(0.94, 1.01)	
19–24 years	Referent	Referent	Referent
25–29 years	0.92	(0.90,0.94)	***
30–34 years	0.94	(0.92,0.97)	***
>35 years	1.27	(1.23,1.30)	***
White	Referent	Referent	Referent
Black	1.02	(1.00,1.05)	***
Asians	0.91	(0.86,0.97)	***
Native Americans/ Alaskan Natives	0.96	(0.82,1.12)	
Others	0.94	(0.91,0.97)	***
Area Deprivation index–Q_1_	Referent	Referent	Referent
Area Deprivation index–Q_2_	0.91	(0.89, 0.94)	***
Area Deprivation index–Q_3_	0.84	(0.82,0.86)	***
Area Deprivation index–Q_4_	0.84	(0.82,0.86)	***
***Clinical Factors***			
Hypertension	2.31	(2.25,2.36)	***
Diabetes	1.76	(1.71,1.82)	***
Asthma	1.08	(1.04,1.14)	***
Substance use disorders	0.91	(0.79,1.06)	
Infections	2.55	(2.37,2.75)	***
***Hospital Factors***			
Occupancy Rate	0.91	(0.90,0.92)	***
No NICU	Referent	Referent	Referent
NICU Status	1.17	(1.14,1.19)	***
Obstetrics care–Level 1	Referent	Referent	Referent
Obstetrics care–Level 2	1.12	(1.09,1.15)	***
Obstetrics care–Level 3	1.07	(1.04,1.10)	***
No system membership	Referent	Referent	Referent
System membership	1.27	(1.25,1.30)	***
Not a teaching hospital	Referent	Referent	Referent
Teaching hospital	0.96	(0.93,0.99)	**
Not-for profit hospitals	Referent	Referent	Referent
For-profit hospitals	0.88	(0.86,0.91)	***
Non-federal Government hospitals	1.08	(1.05,1.11)	***

*** p < .01

** p < .05

* p < .1

Our results on hospital characteristics and odds of C-sections were also consistent with prior studies. We found that one percent increase in occupancy rate of the hospital decreases C-section rate by 9% (OR = 0.91, p<0.01), the results consistent with previous study.

[18]. Likewise, as reported in earlier studies, our results indicate that the presence of NICU within the hospital (OR = 1.17, p<0.01) and higher levels of obstetric care were associated with greater odds of getting a C-section [21]. Compared to not-for-profit hospitals, for-profit hospitals were associated with lower odds of getting a C-section (OR = 0.88, p<0.01). Also, academic hospitals were less likely to be associated with higher C-section rate relative to non-academic hospitals (OR = 0.96, p<0.01).

### Decomposition of the difference in C-section rates across insurance coverage

To investigate if the sociodemographic, clinical, and hospital characteristics of privately insured mothers accounts for their higher risk of getting C-sections, we conducted a decomposition of the differences between publicly insured mothers and those covered by private insurance, the results of which are presented in Table 3. The raw difference in C-section rates is 4.4 percentage points, meaning privately insured women are 4.4 percentage points more likely to get a C-section than women on Medicaid. The decomposition results show that the socio-demographic, clinical, and hospital characteristics between mothers who are privately insured and those on Medicaid account for 33.7% of the difference. Of this explained variance, sociodemographic factors contribute nearly 62.9%, clinical factors contribute 12.3% and hospital-level factors contribute about 24.8%. However, our results indicate that a large portion of the variance remains unexplained (66.3%). In sociodemographic factors, maternal age and socioeconomic status are the major contributors in explaining the variance.

**Table 3:** Decomposition of the differential in NTSV Low risk C-section rates between publicly and privately insured mothers.

	% *pt diff*	% ${\bar{Y}}^{\mathrm{pub}}‐{\bar{Y}}^{\mathrm{pri}}$	z-stat	
${\bar{Y}}^{\mathrm{pub}}‐{\bar{Y}}^{\mathrm{pri}}$	-0.044			
Unexplained	-0.029	66.28		
Explained	-0.014	33.72		
Sociodemographic Factors (Total)		21.2		
Maternal Age	-0.003	7.33	-5.44	***
Race				
White(ref)				
Black	-0.000	0.68	-0.97	
Asians	0.000	-1.12	4.15	***
Native Americans/ Alaskan Natives	-0.000	0.0	-0.29	
Others	-0.000	1.2	-7.35	***
ADI	-0.005	13.12	-15.11	***
Clinical Factors (Total)		4.10		
Hypertension	-0.001	2.78	-29.88	***
Diabetes	-0.0004	0.94	-11.95	***
Asthma	0.000	-0.16	1.53	
Substance use disorders	-0.000	0.16	-1.65	
Infections	-0.0001	0.38	-13.53	***
Hospital Factors (Total)		8.40		
Occupancy Rate	-0.000	1.2	-8.20	***
NICU Status	-0.002	5.9	-13.09	***
Obstetrics care–Level 1 (ref)				
Obstetrics care–Level 2	-0.001	2.96	-4.44	***
Obstetrics care–Level 3	-0.0003	0.85	-1.87	*
System Membership	-0.0002	0.5	-3.91	***
Teaching Status	0.000	-0.36	0.63	
Not-for-Profit (ref)				
For-Profit	-0.0005	1.25	-4.53	***
Government	0.001	-3.9	8.56	***

*** p < .01

** p < .05

* p < .1

Results are presented as marginal effects, with robust standard errors in parentheses using the public (pub) coefficients as the reference. Using the pooled coefficients, results in the explained component variance increased to 37.4%.

## Discussion

C-section, as a life-saving procedure, is performed in situations where vaginal delivery is not possible or when there exist substantial health risk(s) for mothers and/or infants. However, unnecessary use of C-sections can pose a danger to both mother and offspring, especially in low-risk births where C-sections may not be medically necessary. The purpose of this paper was to ascertain if low-risk C-section rates varied based on maternal insurance status, and if rate differences could be attributed to differences in sociodemographic, clinical, and hospital-level characteristics. Using data on live hospital births in Florida, our study found that statistically significant differences exist in the C-section rates between women covered by Medicaid and those covered by commercial, private insurance. Overall, our results are consistent with earlier studies [12, 15].

Our decomposition analysis found that sociodemographic, clinical, and hospital-level factors together explained about 33.7% of the difference in C-section rates across maternal insurance types, with the majority being attributed to sociodemographic factors. Socioeconomic status of the women as measured by ADI was one main contributor in explaining the variance. Consistent with prior studies, our analysis found that advanced maternal age was the major contributor that explained the differences in C-section rates between privately insured women and those covered by Medicaid [26, 28]. Women on private insurance tend to be older, possibly due to higher education and employment, and also more likely to get C-sections to avoid birth complications [26, 29]. Complications associated with advanced maternal age were counterbalanced to some extent by the inclusion of only low risk births in this study; however, a higher prevalence of C-sections among women covered by private insurance persisted. Besides maternal age, clinical factors such as diabetes, hypertension, antepartum bleeding, and substance use disorders in the mothers explain about 12.3% of the differences between the two maternal groups. Our results are in line with a recent study that used decomposition analysis to explain the differences in emergency and elective C-section rates among nulliparous women in public hospitals in Ireland [26]. However, previous research did not differentiate between high risk and low-risk births and only accounted for hospital teaching status as a hospital-level factor. In our study, we found that hospital-level characteristics like advanced obstetric care and presence of a NICU accounted for about 24.8% of explained differences in C-section rates between the insurance groups. Hospitals that offer advanced obstetric care and that have a staffed NICU may be more attractive options for providers who anticipate birth complications. Private insurance plans may also offer greater latitude in terms of the post-partum care that may be covered (e.g., longer infant stay in NICU when necessary) compared with Medicaid coverage. These factors may be responsible, in part, for driving some of the higher C-section rates among privately insured patients since private insurers reimburse at a higher rate than public payors. Our study also found that hospitals that are part of a larger health system were associated with higher odds of C-sections. The association of academic hospitals and C-sections has been established in the literature and our results concur with them [16]. Hospitals that are a part of a larger system may have system-wide practice patterns in place to encourage more resource-intensive procedures and thereby contribute to the bottom line. Targeted interventions to address the high C-section rates in these hospitals may require modifications in overall organizational culture and physician behavior.

A salient finding from our study is that a substantial proportion (66.3%) of the public–private difference in C-section rates remains unexplained. For example, our study failed to capture some modifiable factors such as women’s education, body mass index (BMI), and antenatal visits, all of which have been shown to be associated with the use of C-sections (26), [30]. The exclusion of these critical factors may contribute to some of the unexplained differences. Another possible explanation for the unexplained variance may be behavioral differences among privately insured women related to personal and cultural values which could drive their preferences for the nature of their labor and delivery experience. In the US, 3% of all births are C-section deliveries on maternal request and the obstetric/gynecology providers may be inclined to follow patient requests [31] despite consistent efforts to educate the patient on the pros and cons of C-sections. In addition to patient preference, diagnostic skill differences on the part of obstetricians may also be a critical factor contributing to some of the unexplained differences. Changing provider behavior and improving diagnostic skills could reduce C-section rates by up to 11.7% among the low-risk births [32]. Finally, one hospital-level factor that was not captured in our study was hospital profits per C-section procedure, since hospital profitability has been associated with higher odds of delivering newborns through C-section births [33]. The inclusion of some or all these factors would increase the explained variance if these factors were more prevalent in women covered by private insurance.

Our findings have potentially important practice and policy implications. A target set by Healthy People 2020 is to reduce C-section rates in the US to 24.7%. Several hospital-level strategies such as adhering to guidelines for use of C-sections, and improving physician-provider communication, physician behavior, patient education on vaginal births after C-sections (VBACs) and decision-making around childbirth care have been recommended to meet this goal [34–36]. Hospitals can participate in state/federal programs that are aimed at reducing C-section rates [37]. Additionally, hospital management can put in efforts to change hospital culture, physician practice patterns, and training needs in order to curb unnecessary C-sections. Along with hospital-level efforts, several state policies have been introduced to address C-section rates such as adjusting financial incentives in the form of reduced payments for unnecessary C-sections or models of care that involve a multi-disciplinary team, and also enhancing data collection efforts to examine and address care inequities [38]. According to the Maternal Health 2020 report, hospitals and health systems can provide maternal care and planned childbirth services in areas where there are lack of obstetric services using telemedicine. Timely consultation using telemedicine services and safe transportation of expectation mother to appropriate maternal care can reduce C-section associated with emergency care [17]. States can also encourage changes in workforce and give more practice authority to mid-wives. Use of certified birth attendants like midwives has been linked to lower C-section rates [39].

### Limitations

Our results should be considered in light of several limitations. First, our study focuses exclusively on the state of Florida, and our findings may not be generalizable across the US. The malpractice law and broader medical practice culture in Florida tend to be distinctive to this region and are not necessarily representative of the rest of the US. Second, Florida has very high malpractice premiums for providers. These pressures may have an influence on provider behavior, which has not been captured in our study. Third, we do not account for the nested relationship (physicians within hospitals) in our sample that may affect the impact of hospital factors on our outcome. Physician behavior practices may be a result of differences in their specialty training. Furthermore, our data did not contain clinical details that formed the basis for use of C-section or hospital-level guidelines or policies that warrant the use of C-section.

## Conclusion

Previous studies have established how insurance coverage of an expectant mother influences the use of C-section as a procedure for childbirth. Although C-sections are a medically critical and often necessary procedure, there are reports of its overuse, especially in women covered by private Insurance. In this study, we quantify C-section rates among publicly (Medicaid) and privately insured expectant mothers to determine which factors individual and hospital-level factors contributed most to the differences. Using data from hospital discharges in Florida, covering almost 80% of singleton nulliparous births, we find that around 33.7% of the differences in C-section rates among the two Insurance groups are explained, and most of the difference is explained by certain sociodemographic factors, especially socioeconomic status and maternal age. Although hospital-level factors explain some part of the differences (24.8%) in the explained variance, standardization of hospital practices can only go so far in reducing the inequalities between the Insurance groups. To make U.S. ‘the safest country to give birth in’, further research that investigates additional factors which explain the C-section rate differential between the two insurance groups is required.
